# Digoxin Toxicity in Renal Failure: Resolution With Plasma Exchange After Fab Therapy Failure

**DOI:** 10.7759/cureus.96841

**Published:** 2025-11-14

**Authors:** Jeremy M Williams, Pramod Reddy

**Affiliations:** 1 Internal Medicine, University of Florida College of Medicine – Jacksonville, Jacksonville, USA

**Keywords:** cardiac glycoside, continuous renal replacement therapy (crrt), digoxin toxicity, junctional bradycardia, plasma exchange therapy

## Abstract

Digoxin toxicity is a serious clinical concern, particularly in patients with chronic kidney disease (CKD), due to impaired renal clearance and the potential for both chronic tissue accumulation and acute overdose. Standard therapy with digoxin-specific antibody fragments (Fab) may be insufficient in cases with large tissue digoxin burdens and in patients with acute renal failure, as the delayed clearance of digoxin-Fab complexes can result in complex dissociation and rebound toxicity. We present a unique case of a 73-year-old woman with acute-on-chronic digoxin toxicity in the setting of acute renal failure who exhibited persistent toxicity symptoms despite Fab therapy. The patient underwent plasma exchange (PLEX) to remove lingering digoxin-Fab complexes and remaining free digoxin, resulting in successful clinical resolution and no disease recurrence. This case highlights the use of PLEX as a potential salvage therapy for patients with chronic digoxin toxicity and renal impairment who exhibit persistent toxicity symptoms despite treatment with Fab therapy.

## Introduction

Digoxin toxicity remains a significant clinical challenge, particularly in elderly patients and those with renal impairment, due to its narrow therapeutic index and complex pharmacokinetics [1]. Chronic kidney disease (CKD) not only increases the risk of progressive digoxin accumulation in tissue stores but also impairs renal clearance, setting the stage for acute or chronic toxicity when additional factors, such as medication errors, occur [2]. The clinical presentation of digoxin toxicity is often nonspecific, and laboratory values may not reliably reflect the severity of toxicity, necessitating a high index of suspicion and careful clinical assessment [3,4]. 

The mainstay of treatment for severe digoxin toxicity is the administration of digoxin-specific antibody fragments (Fab), which rapidly neutralize free plasma digoxin and are associated with improved outcomes in most cases [5]. However, in patients with a large tissue digoxin burden and impaired renal function, Fab therapy may be insufficient. The rapid neutralization of plasma digoxin by Fab can alter the plasma-tissue concentration gradient, promoting the redistribution of digoxin from tissue stores into the plasma [5,6]. This process may contribute to the persistence of toxicity symptoms even after treatment with Fab antibody. Furthermore, renal dysfunction delays the clearance of digoxin-Fab complexes, increasing the risk of persistent or rebound toxicity as these complexes gradually dissociate over time [2,6]. 

Conventional extracorporeal therapies such as hemodialysis are ineffective for removing digoxin or its antibody complexes due to their large molecular size and high volume of distribution [7]. Plasma exchange (PLEX) facilitates the clearance of both free and antibody-bound digoxin. It has subsequently emerged as a potential salvage therapy in Fab-refractory cases, particularly when renal excretion is compromised and the risk of rebound toxicity symptoms is elevated [8,9]. 

We describe a case of acute-on-chronic digoxin toxicity in a patient with acute renal failure, unresponsive to Fab therapy, in whom PLEX was successfully used to treat persistent symptoms and prevent recurrent toxicity. This case highlights the importance of understanding digoxin pharmacokinetics in the setting of renal dysfunction, the limitations of standard therapies, and the potential role of PLEX as a life-saving intervention in Fab-refractory digoxin toxicity. 

## Case presentation

A 73-year-old woman with a history of paroxysmal atrial fibrillation, rheumatoid arthritis, CKD, chronic pain, and hypertension presented with 12 hours of progressively worsening generalized weakness. She reported diffuse body aches, abdominal cramping, blurred vision, and persistent nausea, with two episodes of non-bilious, non-bloody vomiting two hours before arrival. She denied chest pain, palpitations, syncope, shortness of breath, numbness, or tingling. Home medications included digoxin 125 mcg daily, apixaban 5 mg twice daily (BID), methotrexate 2.5 mg daily, metoprolol succinate 50 mg daily, oxycodone 10 mg BID as needed, and gabapentin 100 mg BID. 

**Figure 1:**
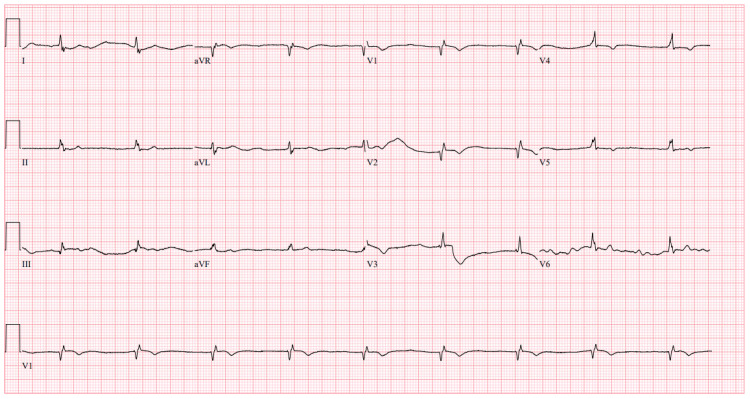
Initial ECG Initial electrocardiogram (ECG) obtained upon arrival to the emergency department (ED) demonstrating junctional bradycardia with a ventricular rate of 54 beats per minute (BPM) and no signs of acute ischemia.

On arrival, vital signs were notable for bradycardia (heart rate 48 bpm); blood pressure, temperature, and oxygen saturation were normal. Physical examination revealed a soft, non-distended but diffusely tender abdomen, muscle strength 2/5 in all extremities, and bradycardia with a regular rhythm on cardiac auscultation. Lung sounds were normal, with no peripheral edema or focal neurologic deficits. Laboratory evaluation (Table 1) showed acute kidney injury (creatinine 3.84 mg/dL; baseline 1.2 mg/dL), hyperkalemia (potassium 6.5 mmol/L), and elevated digoxin level (2.4 ng/mL). High-sensitivity troponin, N-terminal pro-brain natriuretic peptide (NT-proBNP), and creatine kinase were normal. ECG demonstrated junctional bradycardia (ventricular rate 54 bpm) without acute ischemic changes (Figure 1). The patient later disclosed she had mistakenly taken three digoxin tablets that morning, confusing them for pain medication. 

**Table 1:** Patient's laboratory investigations at presentation with reference ranges for comparison NT-proBNP: N-terminal pro-brain natriuretic peptide

Laboratory Investigation	Patient Value	Reference Range
Creatinine	3.84 mg/dL	0.51 - 0.96 mg/dL
Potassium	6.5 mmol/L	3.4 - 4.5 mmol/L
Digoxin level	2.4 ng/mL	0.8 - 2.0 ng/mL
High-sensitivity troponin	8 ng/L	<14 ng/L
NT pro-BNP	87 pg/mL	0 - 125 pg/mL
Creatine kinase	32 U/L	22 - 195 U/L

**Figure 2:**
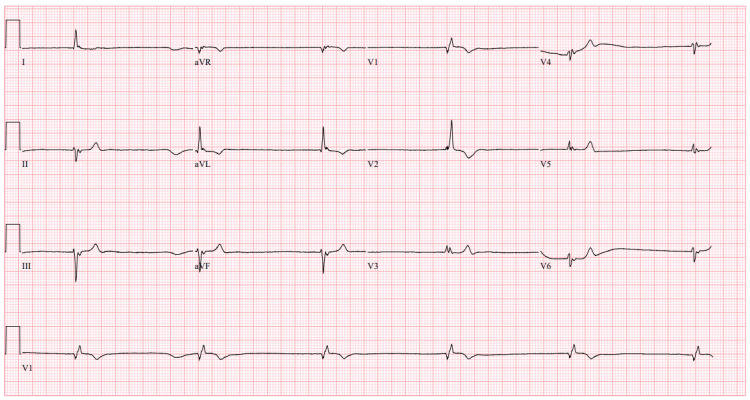
Repeat ECG Repeat electrocardiogram (ECG) obtained one hour after initial treatment demonstrating worsening junctional bradycardia with a ventricular rate of 36 beats per minute (BPM) and no signs of acute ischemia.

**Figure 3:**
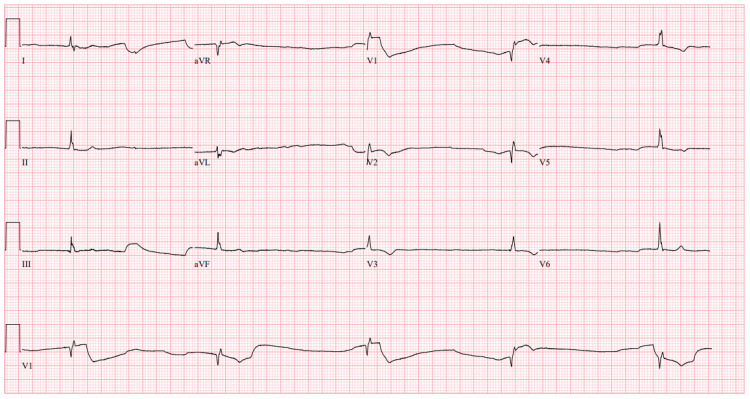
Repeat ECG #2 Repeat electrocardiogram (ECG) obtained at hour two demonstrating further worsening of junctional bradycardia with a ventricular rate of 30 beats per minute (BPM) prompting the need for more invasive intervention.

She received two vials of digoxin-Fab for suspected toxicity, insulin/dextrose for hyperkalemia, and 2 g IV calcium gluconate for myocardial stabilization. One hour later, her bradycardia worsened (ventricular rate 36 bpm) (Figure 2) and hyperkalemia persisted (potassium 6.1 mmol/L). Two additional vials of digoxin-Fab and another round of insulin/dextrose were administered. After another hour, she remained bradycardic (30 bpm) (Figure 3); repeat laboratory results showed digoxin levels of 7.78 ng/mL and potassium at 6.9 mmol/L. Of note, serum digoxin levels on laboratory results may be falsely elevated after administration of Fab due to assay interference, thus further toxicity was graded on clinical signs and symptoms. A temporary hemodialysis catheter was placed, and continuous renal replacement therapy (CRRT) was initiated for refractory hyperkalemia. Due to persistent symptoms of digoxin toxicity despite Fab and CRRT, PLEX was planned but delayed by almost 10 hours due to staffing. CRRT improved hyperkalemia, but bradycardia and symptoms persisted. 

**Figure 4:**
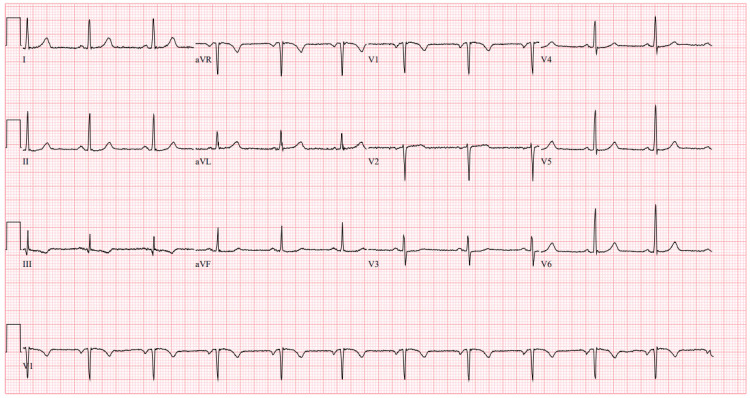
ECG following PLEX Electrocardiogram (ECG) obtained following treatment with plasma exchange (PLEX) showing a resolution of previous junctional bradycardia, now showing normal sinus rhythm with a rate of 71 beats per minute (BPM) and no signs of acute ischemia.

**Figure 5:**
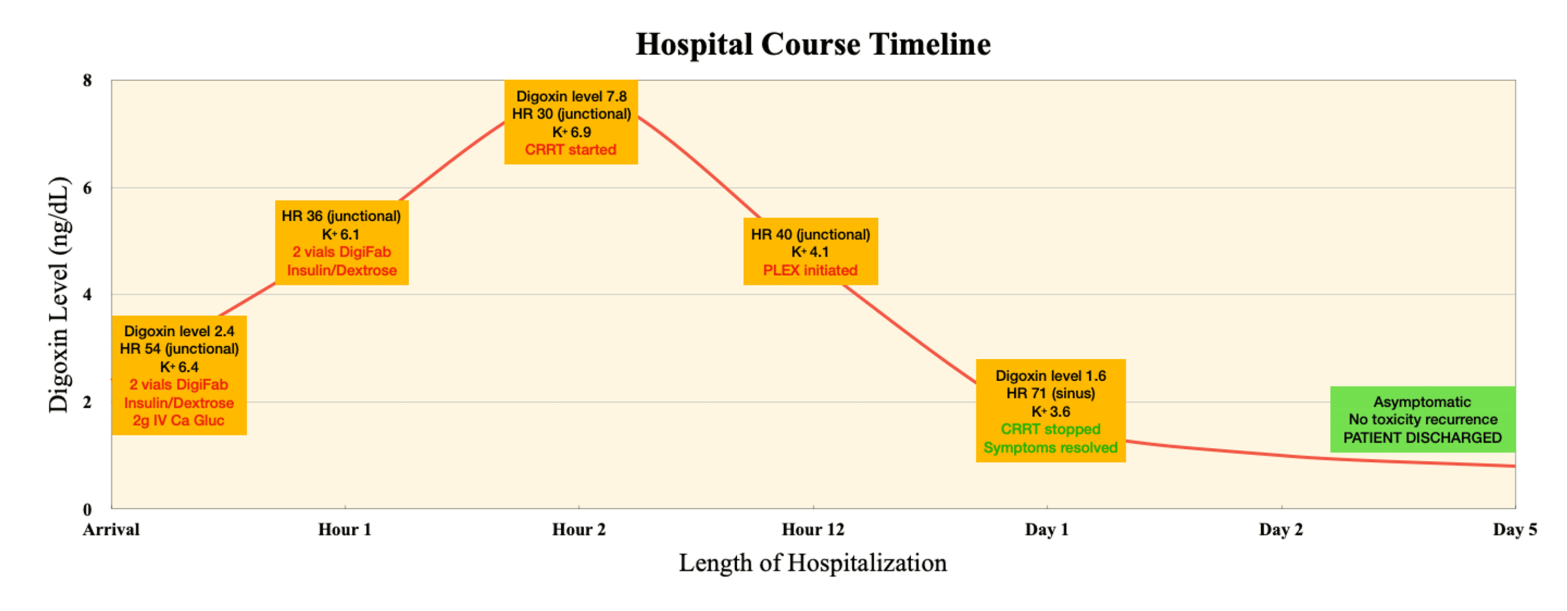
Timeline summarization of hospital course with relevant findings and corresponding interventions Pertinent findings (shown in black) with interventions (shown in red). HR (heart rate displayed in BPM) followed by the corresponding cardiac rhythm shown in parentheses. CRRT: continuous renal replacement therapy, PLEX: plasma exchange

Following PLEX, the patient’s clinical status improved significantly. By the next day, a repeat ECG showed return to normal sinus rhythm (Figure 4), renal function normalized, and the temporary dialysis catheter was removed. Digoxin levels returned to normal, and symptoms resolved. She was discharged after medication education and remained well at one-month follow-up, with no recurrence of toxicity. However, it is important to maintain consistent follow-up as the risk of disease recurrence varies significantly based on individual patient characteristics, such as renal dysfunction. A full timeline of events is summarized in Figure 5. 

## Discussion

This case underscores the unique and complex pathophysiology of acute-on-chronic digoxin toxicity in a patient with renal impairment. It highlights the utility of PLEX as a salvage therapy to treat persistent toxicity symptoms and prevent recurrence when standard treatments fail. CKD predisposes patients to chronic digoxin accumulation in tissue stores due to impaired renal clearance. This occurs as chronically elevated plasma digoxin concentrations promote a redistribution into tissue stores to achieve equilibrium, resulting in a substantial tissue digoxin burden [5,6,10]. In our patient, an acute overdose occurred on top of this chronic tissue burden, leading to severe toxicity. 

The administration of digoxin-specific antibody fragments (Fab) is the cornerstone of therapy for life-threatening digoxin toxicity, as Fab rapidly neutralizes free plasma digoxin and is associated with clinical improvement in most cases [5]. In patients with a significant tissue digoxin burden, Fab therapy effectively reverses the tissue-plasma concentration gradient, mobilizing stored digoxin back into the circulation. This phenomenon can result in persistent or recrudescent toxicity, particularly when renal dysfunction impairs the clearance of both free digoxin and the digoxin-Fab complexes [5,6,11].

In this context, our patient’s persistent symptoms despite multiple doses of Fab and supportive measures can be explained by ongoing redistribution of digoxin from tissue stores and delayed elimination of digoxin-Fab complexes due to renal failure. Conventional extracorporeal therapies, such as hemodialysis, are ineffective for removing these complexes due to their large molecular size and high volume of distribution [7]. The use of PLEX in our case was therefore not only aimed at removing any remaining free digoxin, but more importantly, at facilitating the clearance of circulating digoxin-Fab complexes. By doing so, PLEX helped prevent the recurrence of toxicity symptoms that could arise from the gradual dissociation of these complexes over time in the setting of impaired renal function. Several recent case reports and reviews have demonstrated that PLEX can facilitate the removal of digoxin-Fab complexes and improve clinical outcomes in patients with digoxin toxicity and renal impairment who are unresponsive to standard Fab therapy, supporting its consideration as a salvage intervention in Fab-refractory cases [9,12-14]. However, the evidence remains limited to case reports, and further research is needed to establish optimal timing and efficacy of PLEX in this setting. 

Although the evidence for PLEX in digoxin toxicity is limited to case reports and is not currently included in formal guidelines, our experience adds to the growing body of literature suggesting that PLEX may be a valuable intervention in Fab-refractory cases, especially when complicated by CKD and a large tissue digoxin burden [7,9]. This case highlights the importance of recognizing the potential for ongoing redistribution and delayed clearance in patients with renal impairment. Due to this, continued observation and periodic assessment of serum digoxin and electrolyte levels are recommended to promptly detect and manage any recurrence of toxicity even outside of the acute setting [15]. Furthermore, it supports consideration of PLEX as a life-saving therapy to prevent symptom recurrence when standard treatments are insufficient. Early multidisciplinary evaluation and individualized management are essential for optimizing outcomes in high-risk patients with digoxin toxicity. 

## Conclusions

This case illustrates the challenging management of acute-on-chronic digoxin toxicity in a patient with renal impairment. Standard treatments, such as digoxin-specific antibody fragments (Fab) and renal replacement therapies, were ineffective likely due to a significant tissue digoxin burden and impaired clearance of digoxin-Fab complexes. The successful application of PLEX as a rescue therapy effectively prevented the recurrence of toxicity symptoms by facilitating the removal of circulating digoxin-Fab complexes. This emphasizes the necessity for individualized and multidisciplinary care, suggesting that PLEX may be a valuable, life-saving intervention for certain patients when conventional treatments fail. While PLEX is not indicated in current guidelines, its use remains off-label. Further research is required to better define the indications, timing, and effectiveness of PLEX in managing digoxin toxicity, especially among populations at high risk for persistent or recurrent toxicity due to underlying renal impairment.
